# Metastatic carcinoma of the lung presenting as jugular venous thrombosis: a valuable clinical lesson

**DOI:** 10.25122/jml-2020-0061

**Published:** 2021

**Authors:** How Kit Thong, Vikram Sugumaran, Hafiz Bin Mohamad Mahbob, Tengku Mohamed Izam Bin Tengku Kamalden

**Affiliations:** 1. Department of Otorhinolaryngology, Head and Neck Surgery, KPJ Healthcare University College, Nilai, Negeri Sembilan, Malaysia; 2. Department of Otorhinolaryngology, Head and Neck Surgery, Hospital Sultan Ismail, Johor Bahru, Johor, Malaysia

**Keywords:** Metastatic lung carcinoma, Jugular venous thrombosis, deep vein thrombosis, CT – Computed tomography, ENT – Ear, Nose and Throat, DVT – Deep vein thrombosis, IJV – Internal jugular vein (IJV), IJVT – Internal jugular vein thrombosis (IJVT)

## Abstract

Thromboses of the upper extremity and neck are rare and not as commonly seen as lower extremity deep vein thrombosis (DVT). Internal jugular vein thrombosis (IJVT) is a serious condition with a potentially fatal outcome. Jugular vein thrombosis refers to the formation of intraluminal thrombi anywhere from the intracranial part of the jugular vein to the junction between the internal jugular vein (IJV) and subclavian vein. The relationship between malignancy and thromboembolic disorders has been well established, as Trousseau first described it in 1865. Tumor cells are known to promote hypercoagulability by expressing tissue factors that activate clotting cascades and procoagulants while promoting interactions between the tumor cells, platelets, and endothelial cells via different cytokines, tumor antigens, and their immune complexes. We are reporting our encounter with a patient who presented with extensive left internal jugular vein thrombosis as the first presenting sign of primary lung malignancy.

## INTRODUCTION

Thromboses of the upper extremity and neck are rare and not as commonly seen as lower extremity deep vein thrombosis (DVT). Jugular vein thrombosis refers to the formation of intraluminal thrombi anywhere from the intracranial part of the jugular vein to the junction between the internal jugular vein (IJV) and subclavian vein. IJV thrombosis was first recognized in 1912 in the Ear, Nose and Throat (ENT) by Long as a complication of peritonsillar abscess, also known as Lemierre’s syndrome [1–2]. Other risks of jugular venous thrombosis include head and neck infections, neck surgery or trauma, local skin infections, local or distant malignant tumor (Trousseau’s syndrome), central venous catheter placement, intravenous drug abuse, polycythemia, hypercoagulable state, or idiopathic internal jugular vein thrombosis with no identifiable risk factor and cause [2–3]. The relationship between malignancy and thromboembolic disorders has been well established as it was first described by Trousseau in 1865 [4]. Tumor cells are known to promote hypercoagulability by expressing tissue factors that activate clotting cascades and procoagulants while promoting interactions between the tumor cells, platelets, endothelial cells via different cytokines and their immune complexes [5]. Prince *et al*. reported a similar case in which the detection of venous thrombosis led to the diagnosis of cancer [6]. Thus, in patients diagnosed with DVT, a thorough workup is always necessary to identify the primary cancer. We are reporting our encounter with a patient who presented with extensive left internal jugular vein thrombosis as the first presenting sign of primary lung malignancy.

## CASE REPORT

**Figure 1:**
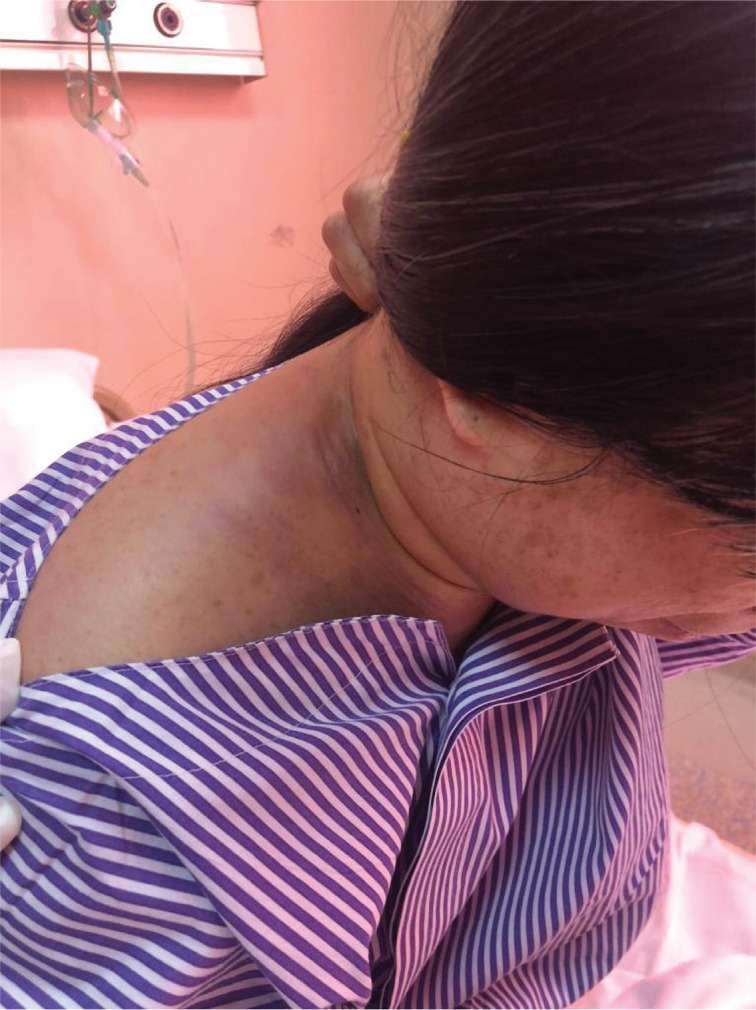
Right neck swelling involving levels II, III, IV as well as the supraclavicular fossa.

**Figure 2:**
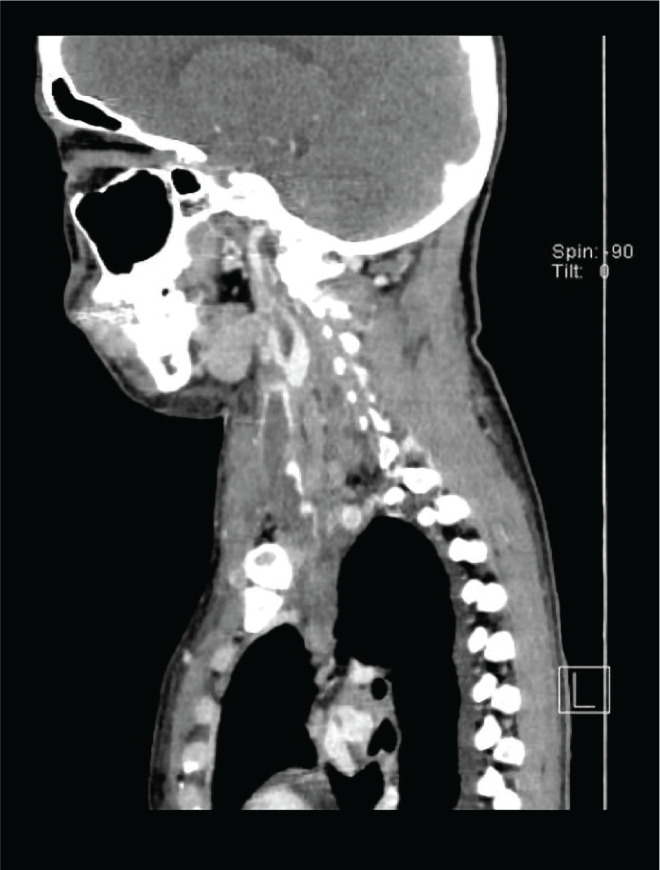
CT scan of the neck (sagittal view): extensive thrombosis of the left internal jugular vein.

**Figure 3:**
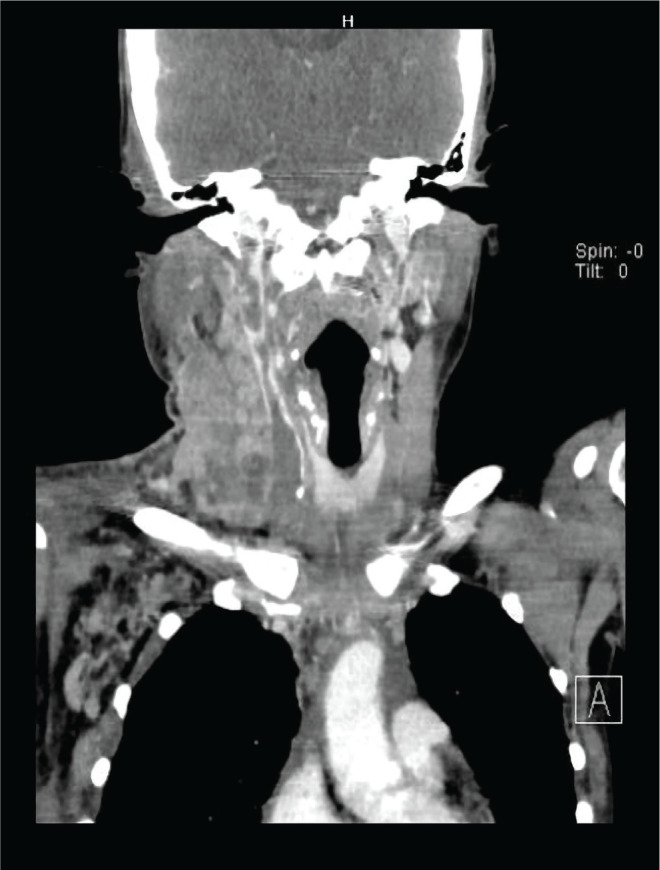
CT scan of the neck (coronal view) showing extensive thrombosis of the right internal jugular vein.

**Figure 4:**
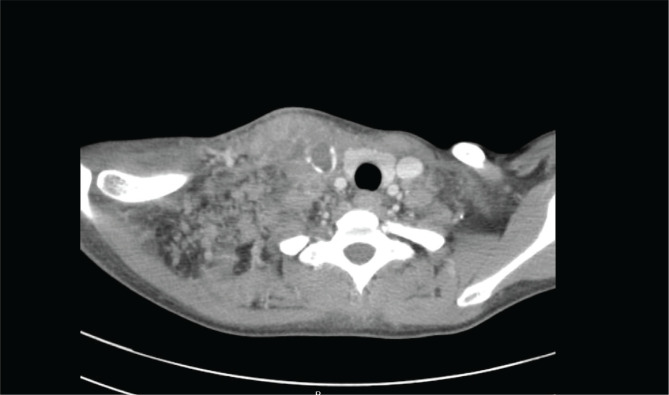
CT scan of the thorax (axial view) with extensive left supraclavicular lymphadenopathy.

A 33-year-old female who was previously healthy presented with swelling and pain over the right side of the neck that had been present for three weeks. The swelling was slowly increasing in size and associated with progressive right arm weakness. She also complained that the pain radiated to the right axilla as well as to the shoulder region. There was no history of fever or night sweats, but she reported losing about 5 kg in weight in the three months prior. She had no cough, and her clinical history was otherwise unremarkable. She had been a chronic smoker for the past 15 years with otherwise no relevant past medical or surgical history. Also, she was not taking any medications prior to the illness. On clinical examination, there was an area of diffuse swelling over the left side of the neck with marked engorgement of the superficial veins and was tender on palpation. The swelling also involved the upper part of the right shoulder (Figure 1). Other than that, the clinical examination was normal. Endoscopic examination of the nasal cavity and larynx was unremarkable. Normal full blood count with a normal erythrocyte sedimentation rate of 4 mm/h and an elevated C-reactive protein of 30 units (normal range is 0–10 units) were found after the initial laboratory investigations. Thrombophilia screening, coagulation profile, urea and electrolytes, thyroid function tests as well as liver function tests were normal. She was referred for an ultrasound examination of the neck, which showed right cervical lymphadenopathy with right IJV and distal right subclavian vein thrombosis. The ultrasound revealed multiple enlarged right cervical lymph nodes involving the II, III, and IV levels. Ultrasound-guided core biopsy of the cervical lymphadenopathy was performed. She initially received intravenous antibiotics and was anticoagulated with low-molecular-weight heparin followed by warfarin. Consequently, she was subjected to computed tomography (CT) of the neck, chest, abdomen, and pelvis. Extensive right IJV thrombosis extending to the right subclavian, right brachiocephalic vein and into the superior vena cava was confirmed (Figures 2 and 3). CT also showed numerous right cervical lymphadenopathies at the II, III, IV, and V levels, and the right supraclavicular region showed extensive mediastinal lymphadenopathies (Figure 4). Chest CT revealed a left lung mass with an irregular margin in the anterior segment of the left lower lobe measuring 2.3 x 2.6 x 2.5 cm. Another lesion with an irregular margin was also seen in the posterior segment of the left lower lobe measuring 1.3 x 1.3 cm with the liver, spleen, pancreas, adrenal glands, and both kidneys appearing normal. The histology of the lymph node core biopsy revealed lymphoid tissue exhibiting malignant tumor infiltrates. The tumor cells stained positive for cytokeratin 7 during immunochemistry, which is suggestive of metastatic carcinomas with possible differentials, including the lung and upper gastrointestinal tract. She was initially prepared for upper aerodigestive tract endoscopy and a CT-guided biopsy of the lung mass; however, the patient refused this procedure. Further investigations to establish a possible primary site, including gynecological and pelvic examinations, proctosigmoidoscopy, and tumor marker studies, were found to be normal. She was referred to the oncology department with a diagnosis of metastatic carcinoma of the lung for further management through chemo- and radiotherapy.

## DISCUSSION

Venous thrombosis mainly occurs in the lower limbs. The veins in the head and neck region are less prone to the development of thrombosis as they are primarily valve-less, and their drainage is aided by the effects of gravity when in the standing position. Upper extremities only account for 4–10% of all cases of DVT [7]. DVT of the IJV has been linked to multiple etiologies, which include central venous catheterization, head and neck infections (such as in Lemierre’s syndrome), malignancy, aneurysm, intravenous drug abuse, and idiopathic and iatrogenic injury [1, 7, 8]. The association between malignancy and venous thrombosis was first described by Armand Trousseau, where migratory thrombophlebitis was found to be associated with gastric malignancy [4]. In malignancy, venous thrombosis is considered a paraneoplastic syndrome; its pathophysiology involves stasis of the regional blood flow due to the effects of tumors encroaching on the blood vessels or compressing the blood vessels or its metastatic lymph nodes. Besides that, blood hypercoagulability and vessel wall damage are also believed to be involved in the process. The hypercoagulable state of malignancy may be attributed to the activation of various clotting factors (e.g., tissue factor [TF], factor Xa, and thrombin), clotting or platelet function inhibitors, and fibrinolysis inhibitors (e.g., plasminogen activator inhibitor), thus promoting thrombus formation [9]. Illuminati *et al*. conducted a retrospective review of 9 patients with internal jugular vein thrombosis related to gastric carcinoma and concluded that the hypercoagulable state in patients with malignancy is multifactorial and might be caused by direct infiltration of circulating tumor cells onto the endothelium of blood vessels; such tumor cells are said to disseminate distally via the hematogenous route [10].

Patients with IJV thrombosis generally present with pain and swelling in the neck region. Tovi *et al*. conducted an extensive series involving patients with septic IJV thrombosis and described the following clinical presentations of the condition: fever (83%), leukocytosis (78%), cervical pain (66%), neck swelling (72%), cord sign (39%), sepsis syndrome (39%), pleuro-pulmonary complications (28%), superior vena cava syndrome (11%), chylothorax (5%), and jugular foramen syndrome (6%) [11]. Pulmonary embolism(PE) is a life-threatening complication of DVT; however, the risks of pulmonary emboli are still poorly understood. Due to the increased risk of PE, patients with a thrombosed IJV are often prescribed anticoagulation medication to prevent clot propagation [1, 3, 6, 8, 9]. Initial examinations should include full blood count, liver function tests, urea and electrolytes, clotting profile, thrombophilia screening, and tumor marker studies, as we did for our patient. Ultrasonography is recommended as the initial diagnostic method for jugular venous thrombosis as it has an average sensitivity of 97% [12]. Currently, venography is rarely performed as it is an invasive procedure and involves both a risk of contrast injection and radiation exposure. Computed tomography scans and magnetic resonance imaging play an equally important role in providing information in regards to the extent of the thrombosis as well as diagnosing and staging the primary malignancy as demonstrated in our patient. Although radiological investigations play an important role in diagnosis, in all cases, both biopsy and upper aerodigestive tract endoscopy are essential for tissue histology and establish a definitive diagnosis.

Medical treatment, which includes initiation therapy with low-molecular-weight heparin followed by warfarin to prevent thromboembolic events, is the primary treatment for jugular venous thrombosis. Other treatment modalities should be considered depending on the etiology of individual cases. Antibiotics at the site of infection, removal of central venous catheters, and appropriate chemo-irradiation for malignant tumors should also be considered. Jugular venous thrombosis due to deep infections at the neck level may require the incision and drainage of abscess collections and local debridement. This case report highlights that the etiology of any deep vein thrombosis should be investigated, and an occult malignancy should not be dismissed.

## CONCLUSION

Jugular venous thrombosis is rare; however, it may be the only manifestation of an occult malignancy and mainly due to the hypercoagulative state of such a patient. This case emphasizes the importance for otolaryngologists to remain vigilant and have a high index of suspicion as malignancies can present atypically with neck swelling and internal jugular vein thrombosis. Early detection of the primary malignancy will improve the prognosis of such patients.
